# Extracellular vimentin is expressed at the rear of activated macrophage-like cells: Potential role in enhancement of migration and phagocytosis

**DOI:** 10.3389/fcell.2022.891281

**Published:** 2022-07-18

**Authors:** Divyendu Goud Thalla, Ashish Chand Rajwar, Annalena Maria Laurent, Johanna Elisabeth Becher, Lucina Kainka, Franziska Lautenschläger

**Affiliations:** ^1^ Experimental Physics, Saarland University, Saarbrücken, Germany; ^2^ Centre for Bioinformatics, Saarland University, Saarbrücken, Germany; ^3^ Centre for Biophysics, Saarland University, Saarbrücken, Germany

**Keywords:** extracellular vimentin, macrophages, activation, migration, polarisation, phagocytosis, vimentin secretion

## Abstract

Macrophages have a vital role in the immune system through elimination of cell debris and microorganisms by phagocytosis. The activation of macrophages by tumour necrosis factor-α induces expression of extracellular cell-surface vimentin and promotes release of this vimentin into the extracellular environment. Vimentin is a cytoskeletal protein that is primarily located in the cytoplasm of cells. However, under circumstances like injury, stress, senescence and activation, vimentin can be expressed on the extracellular cell surface, or it can be released into the extracellular space. The characteristics of this extracellular vimentin, and its implications for the functional role of macrophages and the mechanism of secretion remain unclear. Here, we demonstrate that vimentin is released mainly from the back of macrophage-like cells. This polarisation is strongly enhanced upon macrophage activation. One-dimensional patterned lines showed that extracellular cell-surface vimentin is localised primarily at the back of activated macrophage-like cells. Through two-dimensional migration and phagocytosis assays, we show that this extracellular vimentin enhances migration and phagocytosis of macrophage-like cells. We further show that this extracellular vimentin forms agglomerates on the cell surface, in contrast to its intracellular filamentous form, and that it is released into the extracellular space in the form of small fragments. Taken together, we provide new insights into the release of extracellular cell-surface vimentin and its implications for macrophage functionality.

## Introduction

Macrophages are immune cells that have multiple roles in physiological processes, which range from removal of cellular waste and tissue regeneration and remodelling, to protection against pathogen invasion (Krzyszczyk et al., 2018; Herzog et al., 2019; Zhang et al., 2021). Due to their heterogeneous functions, macrophages have a crucial role in the immune system (Viola et al., 2019).

An earlier study revealed that activation of macrophages by tumour necrosis factor (TNF)-α leads to extracellular cell-surface expression and release of vimentin into the surrounding medium (Mor-Vaknin et al., 2003). Similarly, in atherosclerosis, monocyte chemoattractant protein-1 (CCL2) and oxidised low-density lipoproteins stimulate secretion of vimentin from macrophages (Kim et al., 2020). Vimentin is a type III intermediate filament that is primarily located inside cells of mesenchymal origin. However, vimentin can also be expressed on the outside of cells under conditions such as inflammation, stress and senescence (Mor-Vaknin et al., 2003; Frescas et al., 2017; Patteson et al., 2020). Previous studies have shown that astrocytes, neutrophils, monocytes, apoptotic lymphocytes and endothelial cells can also secrete vimentin (Boilard et al., 2003; Mor-Vaknin et al., 2003; Moisan and Girard, 2006; Greco et al., 2010; Kaplan, 2013; Patteson et al., 2020). Within the cell, vimentin is involved in cellular functions such as adhesion, migration and signalling (Ivaska et al., 2007), while recent studies have indicated its functions as a dynamic extracellular protein in cancers (Satelli and Li., 2011; Satelli et al., 2014; Satelli et al., 2015), viral infections (Yang et al., 2016; Ghosh et al., 2018; Bryant et al., 2006; Schäfer et al., 2017; Suprewicz et al., 2022) and general cellular functions (Thalla et al., 2021). Despite the many functional roles now defined for extracellular vimentin, the characteristics and the circumstances of its secretion remain unclear.

Vimentin that is secreted by activated macrophages is also involved in bacterial elimination (Mor-Vaknin et al., 2003) and the immune response (Ramos et al., 2020). Therefore, we questioned the role of secreted vimentin in macrophage activity, and particularly in phagocytosis. It is already known that activation of macrophages enhances phagocytic activity and improves pathogen clearance (Leopold Wager and Wormley, 2014; Jaggi et al., 2020). It is also known that activation of macrophages by TNF-α results in enhanced phagocytosis of the fungal pathogen *Cryptococcus neoformans* (Collins and Bancroft, 1992)*,* with the same is seen for glial macrophages in response to TNF-α in glial–neuronal cell co-cultures (Neniskyte et al., 2014). However, the role of extracellular vimentin for macrophage activity and functionality is not known.

In this study, we investigated the location and form of this extracellular vimentin. We further examined the influence of extracellular vimentin on macrophage functionality. Using fluorescence microscopy techniques and one-dimensional (1D) patterned lines, we show that vimentin is not equally distributed on the surface of activated macrophages, but is located at the ‘back’ of the cells. We also show that vimentin is released into the extracellular environment in the form of small fragments. Using 2D migration and phagocytosis assays, we further show that addition of recombinant vimentin to macrophages has a similar effect on phagocytic activity and migration as for activated macrophages.

This study thus characterises extracellular vimentin and describes its influence on macrophage functionality. On the basis of these data, we propose a secretion pathway for vimentin. Collectively, these findings are crucial to understand how the immune system is regulated, and they offer new ways to interfere with it.

## Materials and methods

### Cell culture

HL60 cells were cultured in cell culture flasks (Grenier) in RPMI 1640 medium (Gibco) supplemented with 10% foetal bovine serum (Fischer Scientific), 1% 1:1 penicillin-streptomycin (Fischer Scientific) and 1% Glutamax (Gibco). The cells were passaged at a concentration of 10^6^ cells/mL. HL60 cells were differentiated into a macrophage lineage by treatment with 10 nM TPA (Sigma) for 24 h (referred to as macrophages). Post-differentiation, the cells were treated with 5 ng/ml TNF-α (Gibco) for up to 6 days, to induce macrophage activation. Media containing TNF-α was changed every second day for the 3 and 6 days activation.

For confirmation of extracellular vimentin expression, HL60 cells that expressed GFP-tagged vimentin (GFP-Vimentin HL60 cells) were used. These were gifted by Dr. Monika Zwerger, DFG, Germany. These GFP-HL60 cells were cultured as indicated above, with the addition of 0.1 μg/ml puromycin (Gibco) to the growth medium.

### Immunostaining

Immunostaining of extracellular vimentin was carried out using the Alexa 647 fluorophore conjugated to the anti-vimentin V9 antibody (Santa Cruz Biotechnology). The cells were prepared by washing with phosphate-buffered saline (PBS) once to remove traces of the growth medium, and were fixed with 4% paraformaldehyde (Science Service) for 10 min. The samples were then blocked for 1 h with 3% bovine serum albumin (BSA) in phosphate-buffered saline. Finally, the cells were incubated with 1:200 V9 anti-vimentin antibody for 1 h, and then viewed under a fluorescence microscope.

The cell membrane was stained using a WGA CF 488A (Biotium) or Alexa Fluor 549 conjugated wheatgerm agglutinin (WGA) conjugate (W11262, Invitrogen). Cell membrane staining using WGA was performed in conjunction with the V9 anti-vimentin antibody, with WGA staining by incubation with 1:200 anti-WGA antibody for 10 min, followed by fixing of the cells.

For M1 marker, activated macrophages were incubated with 1:100 anti CD68 antibody Alexa 488 (Santa Cruz Biotechnology) for 1 h.

Live-cell imaging was performed using the CSV anti-cell-surface vimentin antibody (Abnova). For this purpose, the cells were incubated with 1:100 CSV for 1 h prior to microscopy.

For permeabilised and non permeabilised comparison, cells were treated with or without triton x-100 0.2% (v/v) for 10 min prior to blocking with 3% (w/v) BSA and cells were incubated with 1:200 beta actin antibody (Proteintech) for 1 h. After washing with PBS, cells were incubated with 1:500 anti-rabbit 488 secondary antibody. Then samples were incubated with 1:200 V9 anti-vimentin antibody for 1 h.

### Quantification of extracellular vimentin

For confirmation and characterisation of the pattern of extracellular vimentin expression on macrophage activation, activation of GFP-vimentin HL60 cells was used. The macrophages were plated on a micro porous transwell membrane (0.4 µm, Corning). The upper and lower compartments were filled with growth medium supplemented with or without TNF-α. Differentiated, inactivated macrophages were used as the control. The medium was collected from the bottom wells after 1 day and 3 days of activation with TNF-α. The collected medium plated into 96-well plates, and the fluorescence intensities were determined using a plate reader (Tecan M200 Pro), with measurement at 488 nm.

### 1D pattern

To produce 1D patterns we prepared PDMS microchips with channels of 5 µm width and attached them to glass bottom dishes using plasma activation. At the next step, the channels were filled with fibronectin (25 μg/ml, Sigma). The PDMS part of the chips were then pulled off and the glass bottom dishes were washed twice with PBS. Afterwards, cells were placed on these patterns (4,000 cells/µl, working volume 500 µL) and stored in the incubator for 1 h. The patterns were washed once with PBS before cells were fixed and stained for vimentin with the V9 antibody.

### Imaging

Fluorescence images were acquired using inverted microscopes. For polarization experiments, the images were acquired using a Ti-Eclipse (Nikon) equipped with Yokagawa spinning disk. For 1D pattern experiments and phagocytosis experiments, the images were acquired using an Epi-fluorescence microscope (Ti-Eclipse, Nikon). For GFP-Vimentin HL60 cells, confocal images were acquired using a LSM900 with Airyscan 2 (Zeiss). The TIRF images were acquired using a Ti-Eclipse with TIRF (Nikon).

### Scanning electron microscopy

For scanning electron microscopy cells were placed on gridded coverslips and treated for activation and differentiation as described before. Cells were fixed in solution containing 2% (v/v) glutaraldehyde (Merck) and 2% (v/v) PFA (Science Service) diluted in 0.2 M sodium cacodylate buffer (Merck) for 120 min.

The samples were incubated three times with 0.1 g sodium borohydride (Merck) in 10 ml PBS for 10 min before they were stained for vimentin as described before. The images were acquired using a spinning disc as described before. After imaging the cell were again incubated in the fixation solution overnight. Next, the samples were incubated in 0.1% tannic acid (Merck). Cell drying was performed by successively replacing water with ethanol (>99.8%, Fisher Scientific) and ethanol with hexamethyldisilizane (98%, Carl Roth and >99%, Sigma Aldrich). Eventually, the samples were sputtered with 4–6 nm platinum. Images were acquired at 5 kV under high vacuum using FEI Quanta 400 electron microscope. Secondary electrons were detected using an Everhart-Thornley detector.

### Migration assay

To investigate the 2D migration of macrophages after activation and stimulation with 100 ng/ml recombinant vimentin (Prospec), fluorescent images of cell nuclei, which were stained using 250 ng/ml Hoechst for 20 min, and bright-field images were recorded over 30 min with a frame rate of 1 min. The cells were kept at 37°C and 5% CO_2_ during the experiment. Cell trajectories were analysed using the ImageJ plugin Trackmate. For blocking the effect of vimentin in activated macrophages, live cell stain of cell surface vimentin antibody (Abnova) was added for 2 h prior to acquisition of migration data. For each cell, the mean migration speed was calculated as the mean value of the instantaneous speeds in between two successive recorded positions.

### Phagocytosis assay

To investigate the phagocytic activity of macrophages under stimulation with extracellular recombinant vimentin, fluorescently-labelled latex beads were used in phagocytosis assays. The assay protocol was developed following the manufacturer instructions (Phagocytosis Assay kits; IgG FITC; Item No. 500290; Cayman Chemicals). For vimentin treatment, differentiated cells were treated with human recombinant vimentin 100 ng/ml (Prospec) for 24 h and activated macrophages were preincubated with V9 anti-vimentin antibody (Santa Cruz) for 2 h prior to phagocytosis experiment.

For quantification of phagocytosis, the microscopy images obtained were analysed using the ImageJ software (Fiji). The analysis consisted of two steps: first, the cell shape was determined by manually outlining the cell on the bright-field image using the Polygon selections tool. The cell ‘mask’ obtained was then saved in the ROI Manager and layered over the fluorescent image. The mean fluorescent intensity of the beads within the cell area can be measured. Additionally, the background fluorescence in an area without cells was then measured for each image. With this data, the “phagocytic index” was calculated for each condition, as *PI* = *I*_beads_/*I*_background_. The average measurement of bead fluorescence corresponded to the number of fluorescently labelled beads that had been phagocytized by the macrophages, and was thus used to quantify their phagocytic activity.

## Results

### Activated macrophages express cell-surface vimentin in a polarised manner

First, we asked whether vimentin is expressed isotopically on the surface of cells. For this, we differentiated HL-60 cells into macrophages by treating them with 12-O-tetradecanoylphorbol-13-acetate (TPA). After 24 h, these macrophages were activated with TNF-α. Interestingly, TNF-α also induces HL-60 macrophage differentiation (Squinto et al., 1989). However, we used TPA to differentiate our cells.

With permeabilisation of the cell membrane during immunofluorescence staining omitted, this ensured that the images acquired using the fluorescently labelled V9 anti-vimentin antibody only showed vimentin on the surface of the cells.

**FIGURE 1:**
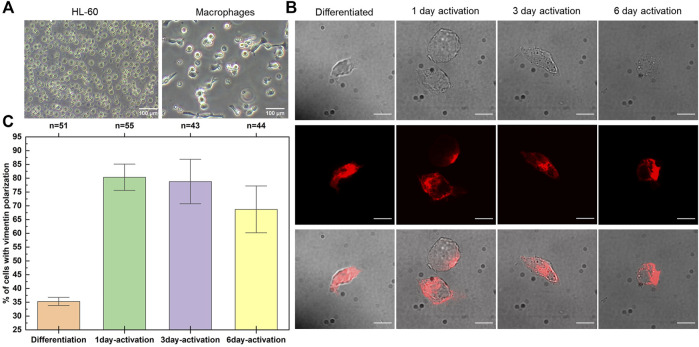
Polarised expression of extracellular vimentin on the surface of TNF-α–activated macrophages. **(A)** Representative HL-60 cells before (left) and after (right) differentiation with TPA. **(B)** Representative non-permeabilised fixed samples of macrophages (Differentiated) stained with the V9 anti-vimentin antibody, showing expression of vimentin on their surface after 1, 3, and 6 days of TNF-α activation. Top row: Phase contrast images. Middle row: Fluorescent images for vimentin (red). Bottom row: Overlay of phase contrast and fluorescent images. Scale bar, 10 µm. **(C)** Quantification of the proportion of macrophages expressing extracellular cell-surface vimentin in a polarised manner over the 6 days of TNF-α activation. n denotes the total number of cells analyzed, error bars correspond to standard deviation.

Upon treatment of the HL-60 cells with TPA for 24 h, they were seen to differentiate into macrophages (Figure 1A). TNF-α treatment of these macrophages triggered the appearance of cell-surface vimentin in a polarised manner, as seen using the V9 anti-vimentin antibody; i.e., the vimentin expressed was not equally distributed over the cell surface. The vimentin expressed on the extracellular surface of these macrophages was instead polarised, as it was predominantly seen over particular areas of the cell surface (Figure 1B). The proportion of the cells that expressed vimentin in this polarised manner was determined by cell counting. There was a >2-fold increase in the polarisation of extracellular cell-surface vimentin in these TNF-α–activated macrophages compared to the non-activated macrophages. During TNF-α activation for up to 6 days, greater proportions macrophages with polarised surface vimentin were seen after 1 day and 2 days (Figure 1C). These data thus show that extracellular cell-surface vimentin is expressed in a polarised manner on these TNF-α–activated macrophages.

**FIGURE 2:**
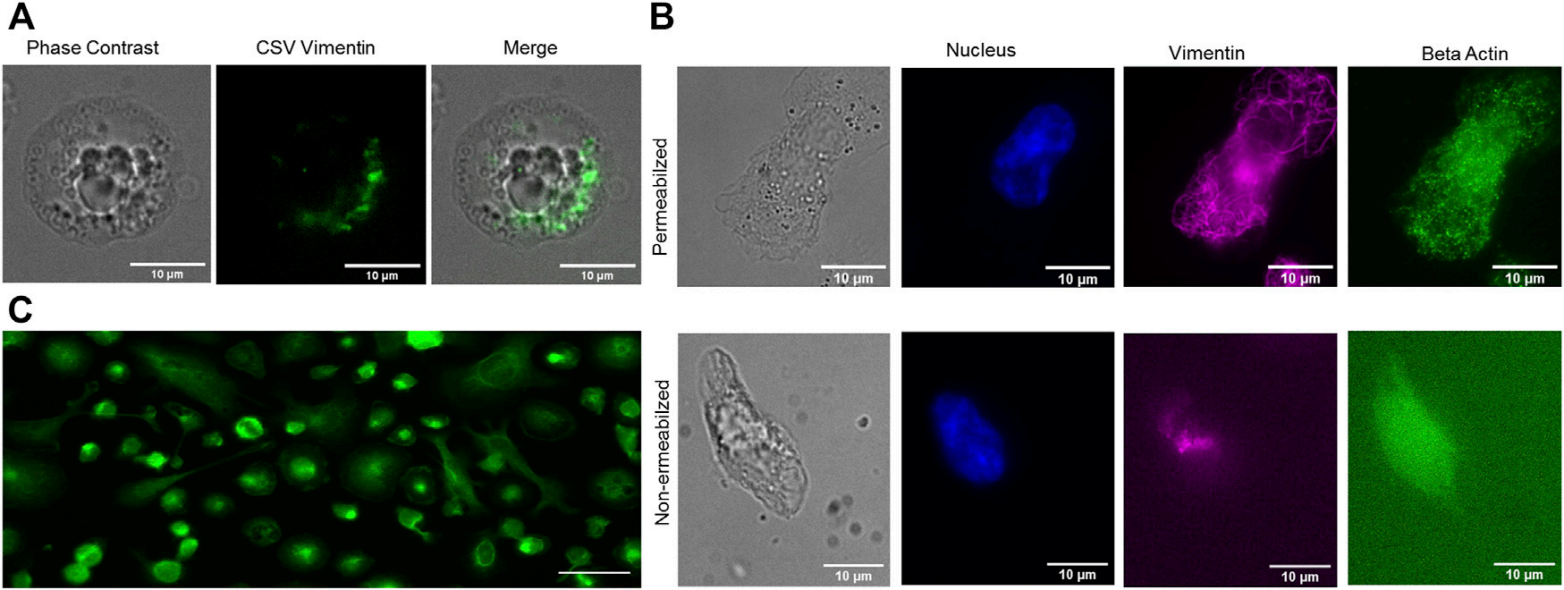
**(A)** Live cell imaging of activated macrophages using CSV antibody. **(B)** Comparison of permeablized vs. non permeablized activated macrophages labelled using beta actin antibody and anti vimentin V9 antibody (Nucleus, blue:actin, green: vimentin, magenta). **(C)** M1 macrophage classification using CD68 macrophage activation marker after TNF- α activation. Scale bar 10 µm.

We further confirm the extracellular localization of vimentin by using CSV antibody which specifically binds to the extracelllular vimentin in live activated macrophages (Figure 2A). This was complemented with comparing the permebalized and non permebalized cells where beta actin was labelled along with vimentin in activated macrophages. Neverthless, some fluorescent signal can be obsereved around the nucleus which could be from the antibody that has been endocytosed. In Figure 2B, it can be clearly seen that fluroscence signal from permeablized activated macrophages is more prominent. However, only unpsecific signal can be observed in non-permeabized activated macrophages. We also classify the macrophages into M1 macrophages upon activation by TNF- α by using CD68 activation marker (Figure 2C).

### Extracellular cell-surface vimentin is predominantly expressed at the back of activated macrophages and secreted in the form small fragments

**FIGURE 3:**
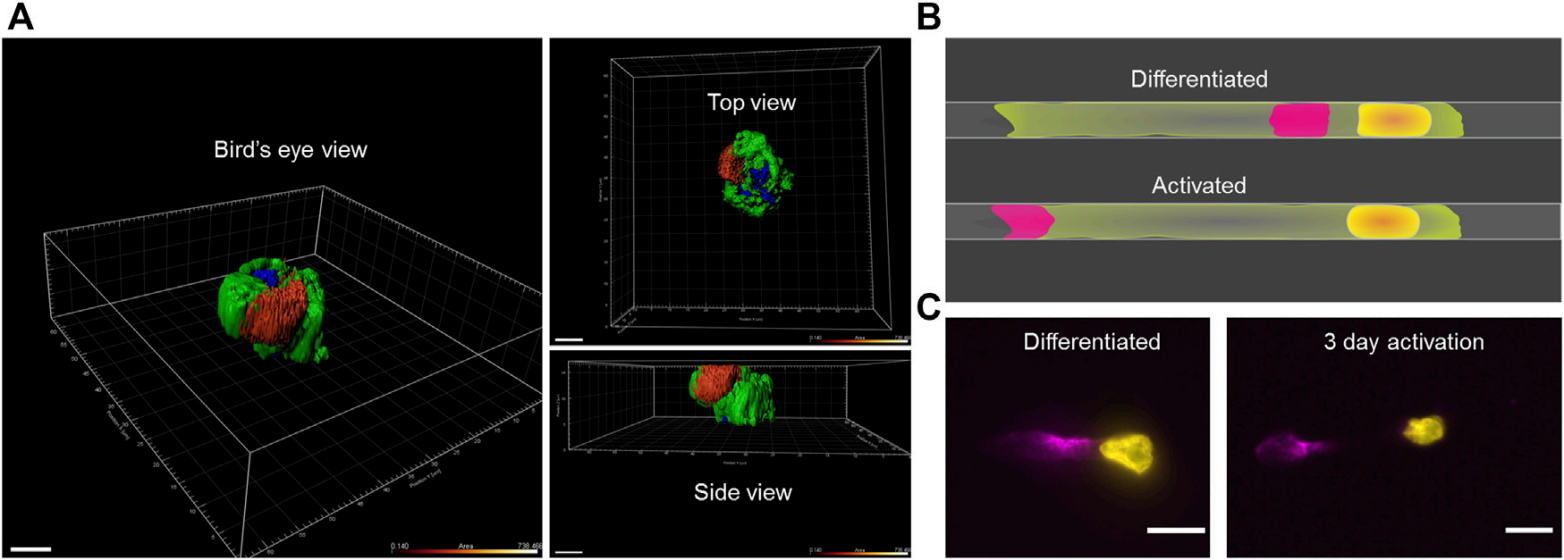
Visualisation of surface vimentin on macrophages patterned on one-dimensional lines. **(A)** Representative three-dimensional projections of a TNF-α–activated macrophage (red, surface vimentin: green, cell membrane), demonstrated using IMARIS. Scale bar, 7 µm. **(B)** Illustration of the vimentin secretion sites (red) for the differentiated and TNF-α–activated macrophages using patterned lines. Yellow, nucleus; green, cell membrane. **(C)** Representative differentiated and TNF-α–activated macrophage attached and elongated along the pattern on a glass coverslip, revealing the site of vimentin secretion (yellow, nucleus; magenta, extracellular cell-surface vimentin). Scale bar, 10 μm.

Although the vimentin was polarised on the surface of these TNF-α–activated macrophages, as the “front” and “back” of these cells were not easily differentiable in these 2D fixed samples, its exact positioning was unknown (Figure 3A). To solve this problem, patterned migration lines on glass coverslips were used, whereby the front and back of the cells can be distinguished by recording time-lapse movies. By following the macrophage migration on patterned lines coated with fibronectin, a simplified cell shape can be defined that allows visualisation of the position on the extracellular cell surface of the vimentin upon TNF-α activation (Figure 3B).

Here, the nucleus was always at the front end of these macrophages during migration. The position of the nucleus was then used as the reference to define the front of the fixed cells. Using this method, the surface vimentin was seen to be polarised at the back of the activated macrophages, allowing us to conclude that vimentin was secreted from the back of these TNF-α–activated macrophages. In contrast, prior to TNF-α activation, the differentiated macrophages were seen to secrete vimentin at a site close to the nucleus (Figure 3C). As, 1 and 3 days activation showed comparable vimentin polarization (Figure 1C), here we used 3 days activation in 1D pattern experiment (Figure 3C).

**FIGURE 4:**
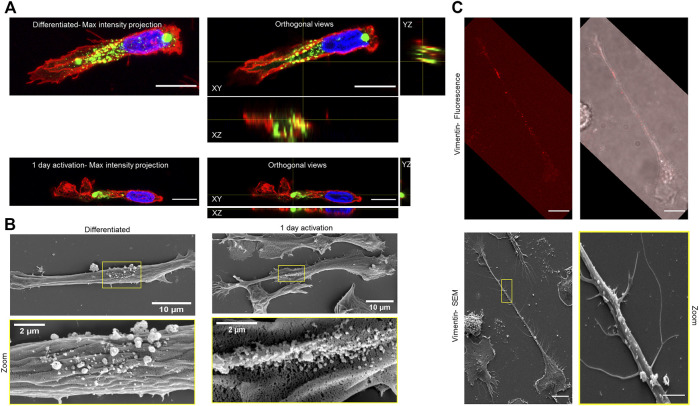
Visualisation of surface vimentin on macrophages differentiated from GFP-vimentin HL60. **(A)** Maximum intensity projection and orthogonal views of differentiated and TNF-α–activated macrophage (red, membrane; green, surface vimentin; blue, nucleus), acquired with confocal microscopy. Scale bar, 10 µm. **(B)** Scanning electron microscopy images of vimentin secretion sites for the differentiated and TNF-α–activated macrophages. Bottom row: higher magnification of vimentin secretion site marked with yellow rectangle. This study was performed on 2D (glass coverslips) not on fibronectin coated 1D patterns. In order to have more residual cells after differentiation and activation, we used 1 day activation of both confocal LSM900 **(A)** and SEM imaging **(B)**. **(C)** Confocal and SEM imaging using coverslips with grid. Top: Fluorescence images of vimentin (red; V9 antibody) stained in non-permeablized activated macropahges. Scale bar 10 μm. Bottom: SEM images of the same cell where the dot like structure can be seen at the same site of vimentin staining. Bottom (Right): magnified image of area marked in yellow of left. Scale bar 2 μm.

To further resolve the structure of the polarized vimentin on the cell surface, we used confocal microscopy for imaging. For this, genetically transformed HL-60 cells with vimentin tagged with green fluorescent protein (GFP-vimentin HL60 cells) were differentiated and activated. Here, images of elongated macrophages were acquired in order to evaluate the structure as well as the position of the surface vimentin. We observed small fragments of vimentin at the secretion sites of both differentiated and TNF-α–activated macrophages (Figure 4A). In order to confirm the small fragments are on the outside of the cell, images of differentiated and TNF-α–activated macrophages were acquired using scanning electron microscopy (SEM) (Figure 4B). Further, we confirmed the presence of vimentin on the dot like structure by visualizing the same cell in both confocal and SEM by using coverslip with grid (Figure 4C).

### Quantification of vimentin secretion upon activation

**FIGURE 5:**
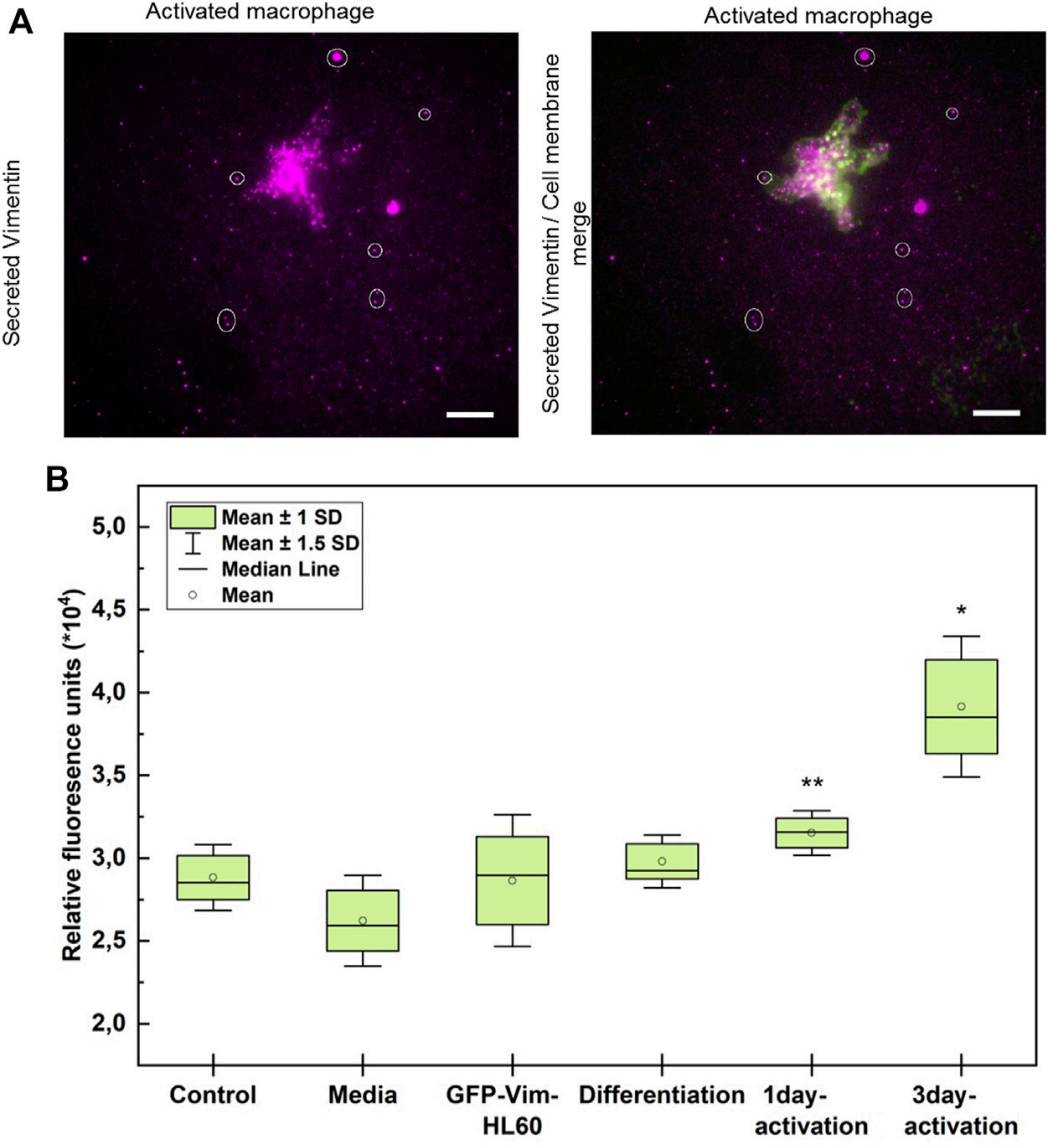
TNF-α–activated macrophages secrete vimentin into the extracellular space. **(A)** Representative total internal reflection fluorescence microscopy of secreted vimentin (magenta) around a TNF-α–activated macrophage (green, cell membrane). Scale bar is 10 µm. **(B)** Quantification of the relative fluorescence of vimentin in the media from activated macrophages expressing GFP-tagged vimentin (repeated 2 times with six replicates of each condition). Conditions: Control, blank; media, RMPI; Differentiation, media from differentiated macrophages; 1 day activation TNF-α; 3 days activation, media from macrophages activated for 3 days with TNF-α. * *p* < 0.05; ** *p* < 0.01 (Student’s t-tests).

It is known that as well as being expressed on the extracellular cell surface, vimentin can then be released into the extracellular surroundings of the cell (Danielsson et al., 2018). We thus asked whether, and in what way, the vimentin secreted by these TNF-α–activated macrophages is functional. Here, we imaged the contact region between the activated macrophages and the glass bottom of the dish to visualise the vimentin released into the medium from the extracellular surface of the activated macrophages, using total internal reflection fluorescence (TIRF) microscopy. Fixed non-permeabilised 3-days-activated macrophages were fluorescently labelled using the V9 antibody. This revealed vimentin agglomerates in the vicinity of the activated macrophages (Figure 5A). These vimentin agglomerates appeared to be in a non-filamentous and fragmented form.

To quantify the vimentin released from these activated macrophages, 0.4-µm transwell insert assays were used with genetically transformed HL-60 cells in which vimentin was tagged with green fluorescent protein (GFP-vimentin HL60 cells). These GFP-vimentin HL60 cells were differentiated using TPA and placed inside the upper chambers of transwell inserts. They were then activated with TNF-α, and left for 1 day and 3 days. The vimentin released into the cell medium passed through the pores of the membrane and was collected in the bottom chamber along with the culture medium. The medium in the bottom chamber was transferred to 96-well plates, and the fluorescence intensity was measured using a plate reader. These data showed that the amount of vimentin released into the medium depended on the time of TNF-α activation of these macrophages (Figure 5B).

### Extracellular vimentin enhances migration and phagocytosis of macrophages

Macrophages are known to have a vital role in the immune system through phagocytosis of cellular debris and elimination of bacterial pathogens. We thus next investigated this extracellular cell-surface vimentin on the functionality of macrophages, in terms of their migration and phagocytosis.

**FIGURE 6:**
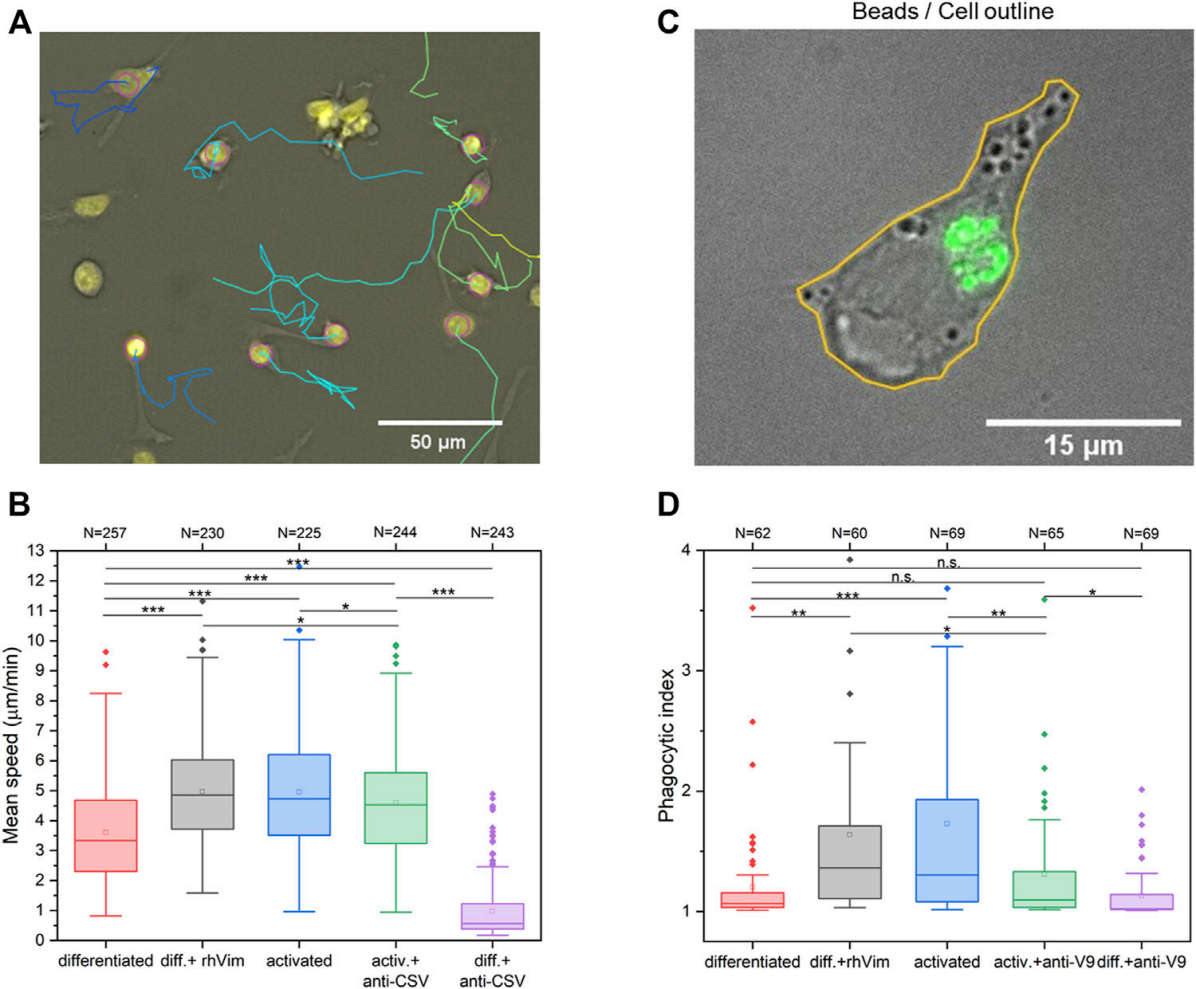
Stimulation of migration and phagocytosis of macrophages by addition of recombinant (rh)Vimentin. **(A)** Migration tracks of TNF-α–activated macrophages visualised using TrackMate ImageJ plugin.**(B)** Quantification of migration speeds of differentiated macrophages without and with addition to the medium of 100 ng/ml rhVimentin and anti-cell-surface vimentin antibody, and of TNF-α–activated macrophages without and with addition of the anti-cell-surface vimentin antibody (CSV). **(C)** Visualisation of fluorescent beads (green) phagocytosed by a TNF-α–activated macrophage using fluorescence microscopy. Cell outline (yellow) defined from phase contrast image. **(D)** Phagocytic index determined by the fluorescence intensity within the differentiated macrophages without and with addition of 100 ng/ml rhVimentin and anti-vimentin antibody (V9), and of TNF-α–activated macrophages without and with addition of the anti-vimentin antibody (V9). *, *p* < 0.05; **, *p* < 0.01; ***, *p* < 0.001. n.s. not significant (Student’s t-tests). All experiments were repeated three times. The data is presented as box plots with whiskers drawn within the 1.5 IQR value. N indicates the total number of cells analyzed.All experiments were done three times.

The migration speeds of the macrophages were measured using a 2D migration assay (Figure 6A). As the media from the TNF-α–activated macrophages that contained secreted vimentin is expected to have some residual TNF-α, recombinant vimentin was used here. Addition of recombinant vimentin to the differentiated macrophages significantly increased their migration speed. Further, the migration speed of the TNF-α–activated macrophages was significantly reduced when they were pre-incubated with the CSV antibody (Figure 6B).

To investigate the phagocytic activity of macrophages under the influence of extracellular vimentin, phagocytosis assays using fluorescently-labeled latex beads were carried out to measure the phagocytic process *in vitro*. With phagocytosis analysed according to the phagocytotic index defined by the intracellular fluorescence intensities following phagocytosis of fluorescent beads (Figure 6C), this was seen to be significantly increased in the TNF-α–activated macrophages compared to the differentiated macrophages (Figure 6D). Further, this effect was mimicked by addition of 100 ng/ml recombinant vimentin to the differentiated macrophages, while it was blocked by the V9 anti-vimentin antibody in the TNF-α–activated macrophages (Figure 6D). As for the migration effect, this enhanced phagocytosis might be due to the high expression levels of vimentin in the TNF-α–activated macrophages. Thus, from these data, we can conclude that extracellular addition of recombinant vimentin enhances both the migration and phagocytosis of these macrophages.

## Discussion

The detection of vimentin in the extracellular space then promoted the question as to how it is secreted from inside these TNF-α–activated macrophages. Previously this extracellular vimentin was thought to have been released from necrotic cells, although it has also been suggested that it might be secreted. Previous studies have shown exosomes as a source of vimentin, and demonstrated that these can transport and release vimentin into the extracellular space (Chen et al., 2016; Adolf et al., 2018; Parvanian et al., 2020). Exosomes are packaged with membranes in the Golgi apparatus, and in activated macrophages, block of transport through the Golgi apparatus inhibits the release of extracellular vimentin (Mor-Vaknin et al., 2003). This has thus strengthened the idea that vimentin is secreted with the help of exosomes. However, it has remained unclear what the characteristics of this secreted vimentin are.

In the present study, we show that vimentin is released from the back of these TNF-α–activated macrophages, and that this polarised release is enhanced by the macrophage activation. Moreover, using confocal, TIRF and SEM, we have confirmed that the structure of the secreted vimentin is not filamentous, as is its intracellular counterpart, but is in form of fragments, as indicated in recent studies (Suprewicz et al., Lalioti et al., 2021). Nevertheless, further confirmation of data from GFP-vimentin HL60 is needed as it may not behave identical to endogenous vimentin.

**FIGURE 7:**
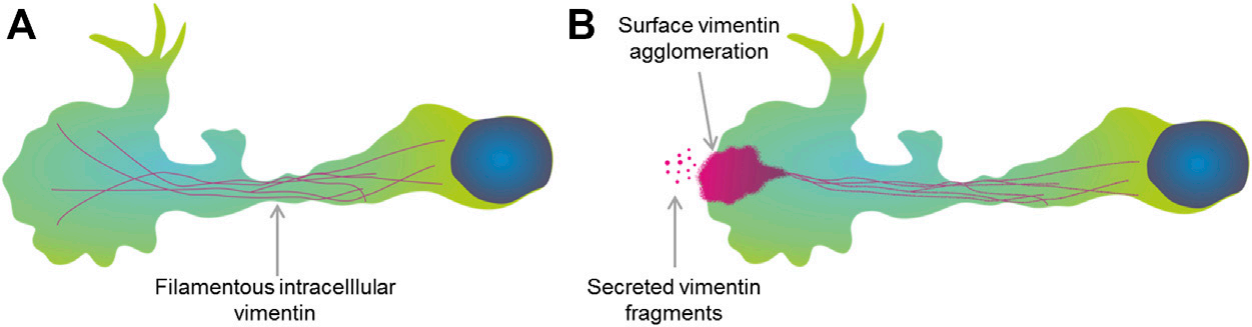
Proposed model of vimentin secretion. **(A)** A macrophage with intracellular vimentin in the filamentous form. **(B)** Dissociation of filamentous vimentin at the cell membrane in the activated macrophage might lead to agglomeration at the surface of the macrophage. Then, the vimentin can be released into the extracellular space in the form of small fragments.

It is believed that post-translational modifications are a prerequisite for vimentin secretion from macrophages and endothelial cells (Noh et al., 2016; Liu et al., 2020; Patteson et al., 2020), which would appear necessary to break down the long vimentin filaments to smaller fragments (Mónico et al., 2019). In the case activated macrophages, the extracellular vimentin was shown to be phosphorylated (Mor-Vaknin et al., 2003). A recent study showed that vimentin is recruited to the cell membrane via an alteration in the filamentous form to an oligomeric form that consists of 4–12 monomers (Hwang and Ise, 2020). This multimeric form of vimentin showed a higher binding affinity to lipid bilayers compared to that of filamentous vimentin. However, the mechanism by which the intracellular vimentin is secreted into the extracellular space is not well characterised. Here, by combining data from the literature and the findings from the present study, we can predict a secretion mechanism as we illustrated in Figure 6. As seen from the present study, extracellular cell-surface vimentin is polarised at the back of TNF-α–activated macrophages, and the images from TIRF shows that small fragments of vimentin either from exosomes or filaments are released close to a large agglomerate of vimentin on the cell surface. Therefore, we propose that at the membrane surface of these TNF-α–activated macrophages, vimentin filaments disassemble into small fragments, to form agglomerates, which can then be released into cell medium or into blood serum (Figure 7B).

Vimentin expression in the extracellular space of cells has been attributed to circumstances such as cell activation, senescence, injury and stress (Mor-Vaknin et al., 2003; Frescas et al., 2017; Walker et al., 2018). In most of these scenarios, the secretion of vimentin is related to immune activity (Ramos et al., 2020). For example, the vimentin secreted by activated macrophages has been suggested to be involved in immune functions via generation of oxidative metabolites and elimination of bacteria (Mor-Vaknin et al., 2003). Another example is shown in patients with rheumatic arthritis, where neutrophils secrete citrullinated vimentin during the release of neutrophil extracellular traps that are produced to immobilise pathogens and promote immune responses (Carmona-Rivera et al., 2013; Kaplan, 2013). Further, in the *Mycobacterium tuberculosis*, vimentin binds to the natural killer cell surface receptor NKp46 and contributes to lysis of infected cells (Garg et al., 2006). Finally, extracellular vimentin blocks pro-inflammatory secretion by activated dendritic cells, which promotes inhibition of adaptive immune responses. This mechanism prevents tissue damage and promotes bacteria elimination (Yu et al., 2018).

On the basis of this evidence that extracellular vimentin partially regulates inflammatory processes, we investigated the role of extracellular vimentin on macrophage function. Using migration assays, we show that elevated levels of extracellular cell-surface vimentin enhance the migration speed of macrophages. Here, it was not relevant if the extracellular vimentin was added as recombinant vimentin or if it was from secretion of activated macrophages. This increase in macrophage migration by addition of recombinant vimentin complements our recent study where we showed similar effects in MCF-7 cells (Thalla et al., 2021).

Earlier studies have demonstrated that increases in phagocytic activity by macrophages involves extracellular vimentin. However, in these previous studies, the vimentin was expressed on the surface of apoptotic neutrophils and T cells and acted as a signalling agent to attract macrophages and further facilitate the elimination process (Boilard et al., 2003; Moisan and Girard, 2006; Ise et al., 2012; Starr et al., 2012). On the surface of phagocytes, vimentin interacts with O-Glc-NAc-modified proteins expressed on apoptotic cells, which generates an “eat me” signal for elimination by macrophages (Ise et al., 2012). To date, surface vimentin has been believed to be a mediator that helps to attract macrophages towards the cells that need to be phagocytosed. However, one study that investigated extracellular vimentin directly expressed on activated macrophages demonstrated enhanced bacteria elimination (Mor-Vaknin et al., 2003). In the present study, we further explored the effects of extracellular vimentin expressed on the surface of macrophages in terms of macrophage function. We show that recombinant vimentin treatment of HL-60 differentiated macrophages has a similar effect as TNF-α–activated macrophages in terms of enhanced phagocytic activity. Interestingly, in a recent study it was shown that extracellular addition of vimentin lead to TNF- α secretion in macrophages (Kim et al., 2020). Consequently, this could be a possible mechanism behind the enhanced migration and phagocytic activity in macrophages.

Taken together, we demonstrate that vimentin is expressed in a polarised form on the surface of activated macrophages, and that it is released in a fragmented form. We also show that extracellular vimentin influences the functionality of macrophages by enhancing their migration and phagocytosis.

Altogether, these data suggests that extracellular vimentin is used to regulate macrophages in the immune system through effective elimination of bacterial pathogens.

## Data Availability

The raw data supporting the conclusions of this article will be made available by the authors, without undue reservation.
